# Construction and investigation of a circRNA-associated ceRNA regulatory network in Tetralogy of Fallot

**DOI:** 10.1186/s12872-021-02217-w

**Published:** 2021-09-14

**Authors:** Haifei Yu, Xinrui Wang, Hua Cao

**Affiliations:** 1 Department of Cardiac Surgery, Fujian Maternity and Child Health Hospital Affiliated to Fujian Medical University, Fuzhou, Fujian People’s Republic of China; 2 Key Laboratory of Technical Evaluation of Fertility Regulation for Non-human Primates, National Health and Family Planning Commission, Fuzhou, Fujian People’s Republic of China; 3 Medical Research Centre, Fujian Maternity and Child Health Hospital, Affiliated Hospital of Fujian Medical University, Fuzhou, Fujian People’s Republic of China

**Keywords:** Disease progression, Gene regulatory networks, RNA, Small untranslated, Tetralogy of Fallot

## Abstract

**Background:**

As the most frequent type of cyanotic congenital heart disease (CHD), tetralogy of Fallot (TOF) has a relatively poor prognosis without corrective surgery. Circular RNAs (circRNAs) represent a novel class of endogenous noncoding RNAs that regulate target gene expression posttranscriptionally in heart development. Here, we investigated the potential role of the ceRNA network in the pathogenesis of TOF.

**Methods:**

To identify circRNA expression profiles in TOF, microarrays were used to screen the differentially expressed circRNAs between 3 TOF and 3 control human myocardial tissue samples. Then, a dysregulated circRNA-associated ceRNA network was constructed using the established multistep screening strategy.

**Results:**

In summary, a total of 276 differentially expressed circRNAs were identified, including 214 upregulated and 62 downregulated circRNAs in TOF samples. By constructing the circRNA-associated ceRNA network based on bioinformatics data, a total of 19 circRNAs, 9 miRNAs, and 34 mRNAs were further screened. Moreover, by enlarging the sample size, the qPCR results validated the positive correlations between hsa_circ_0007798 and *HIF1A*.

**Conclusions:**

The findings in this study provide a comprehensive understanding of the ceRNA network involved in TOF biology, such as the hsa_circ_0007798/miR-199b-5p/*HIF1A* signalling axis, and may offer candidate diagnostic biomarkers or potential therapeutic targets for TOF. In addition, we propose that the ceRNA network regulates TOF progression.

**Supplementary Information:**

The online version contains supplementary material available at 10.1186/s12872-021-02217-w.

## Background

Congenital heart disease (CHD) is the most common congenital malformation, consisting of structural and functional abnormalities of the cardiovascular system that develop during the embryonic period and are present at birth. CHD affects 0.5–0.8% of all live births and is the leading cause of neonatal death [1–3]. According to the changes in cardiac haemodynamics and pathophysiology, CHD can be divided into two main categories: cyanosis and nonmagnetic form. Tetralogy of Fallot (TOF) is the most common type of cyanotic congenital heart disease, accounting for 7–10% of all common CHDs [4, 5]. TOF consists of a ventricular septal defect (VSD), pulmonary stenosis, right ventricular hypertrophy, and aorta overriding the ventricular septum. With surgical correction progressing in recent years since the initial successful repair of TOF, the attention of the research community has shifted towards understanding causation [1, 6]. However, the exact pathogenesis of TOF remains elusive. Numerous studies have shown that miRNA expression should be disordered in TOF heart tissues [7]. To date, there have been few relevant reports on the comprehensive profiling of noncoding RNAs (ncRNAs) in TOF hearts.

Current knowledge and understanding have shown that temporal and spatial expression patterns of heart development-related genes are essential in the regulation of cardiomyogenesis, which means that both genetic and epigenetic factors play a crucial role throughout development [8, 9]. Abnormal functional connectivity in the regulation network leads to failure of cardiac cell lineage specification, commitment, and differentiation [10, 11]. Unfortunately, the underlying molecular mechanisms remain relatively poorly understood. Using next-generation sequencing technology, increasing evidence supports the deregulation of ncRNAs in the dysregulation of cardiomyogenesis [12, 13]. Different types of ncRNAs include microRNAs and a variety of long ncRNAs (lncRNAs), such as lncRNAs, antisense RNAs, pseudogenes, and circular RNAs (circRNAs). For circRNAs, using a high-throughput circRNA microarray is beneficial for detecting and studying circRNAs compared with circRNA-seq [14]. However, microarray can only explore known circRNAs. To identify novel circRNAs, next-generation sequencing technology is a suitably sensitive tool.

Recently, circRNAs, as hot topics and trends in scientific research, have provided further opportunities for better understanding the biological mechanisms of heart diseases [15, 16]. CircRNAs, as closed structures, are evolutionally conserved, tissue-specific, and relatively stable. In addition, accumulating integrative analyses have demonstrated that circRNAs are involved in regulating gene transcription and biological processes by acting as miRNA “sponges”, competing for endogenous RNAs (ceRNAs), including RNA transcripts, miRNAs, and circRNAs. All of the observed characteristic features give circRNAs obvious advantages in the exploration of new clinical diagnostic biomarkers and therapeutic targets [17]. However, there have been only a few reports exploring circRNAs in human TOF, even though an enrichment for various diseases/functions of these promising circRNA findings was identified.

In the present study, we performed microarray analysis and evaluated circRNA expression profiles in heart samples from healthy controls and foetuses with TOF. Moreover, by using bioinformatics analysis of these differentially expressed circRNAs, we screened key circRNAs and constructed a ceRNA network. Our results may contribute to a mechanistic understanding of the pathogenesis and the identification of potential therapeutic targets for TOF in the future.

## Methods

### Participants

All microarray analyses were run on samples from 3 foetuses with nonsyndromic TOF (i.e., no 22q11.2 deletion) and 3 normally developing hearts of foetuses that were ultimately aborted. Additionally, we recruited an additional 20 subjects (10 nonsyndromic TOF patients and 10 healthy controls) to expand the sample size in the independent validation via qPCR.

We obtained foetal hearts (at approximately 150 days of gestation) from the Fujian Provincial Maternity and Children’s Hospital (approval number: IRB NO. 2019–046). The foetal hearts were dissected by a surgeon who also performed many of the reconstructions of the conotruncal defects (Hua Cao) to ensure that the analysed myocardial tissue samples were all taken from a similar location (right ventricle outflow tract) [18]. After each sample was removed, the bloodstains were washed with precooled physiological saline, and the sample was then immediately stored in RNAlater at − 80 °C for subsequent processing.

### RNA extraction and quantitative PCR (qPCR)

Total RNA content was extracted from the tissues using TRIzol Reagent (Invitrogen, CA, USA) and was reverse transcribed into cDNA using the PrimeScript RT reagent Kit (TaKaRa, Tokyo, Japan) according to the manufacturer’s instructions. The relative expression levels of mRNA and circRNA (normalized to β-actin) were analysed by the 2^−ΔCt^ relative quantification method, and the relative circRNA expression was calculated with the 2^−ΔCt^ method [19]. qPCR was performed using a SYBR Green qPCR kit (TOYOBO, Tokyo, Japan) in the StepOne PCR system (Applied Biosystems). Primer sequences are shown in Table 1.

**Table 1:** Primers used for qPCR analysis of circRNA and mRNA levels

Target	Primer Sequence 5` to 3`	
	Forward	Reverse
*HIF1A*	GTCTGCAACATGGAAGGTATTG	GCAGGTCATAGGTGGTTTCT
*ACTB*	GAGAAAATCTGGCACCACACC	GGATAGCACAGCCTGGATAGCAA
hsa_circ_0007798	CCTGGAAGAGATGGATCAGAAA	GCATGCACGGCAGAAATC

### Profiling of circRNA expression by the Arraystar Human Circular RNA V2.0

The Arraystar Human Circular RNA Microarray V2.0 (Arraystar, Inc.), performed by Kang Chen Biotech (Shanghai, China), was designed for the purpose of profiling circRNAs in the human genome. Scanned image processing was analysed using Agilent Feature Extraction Software Version 11.0.1.1. CircRNAs (|fold-change|≥ 2.0 and *P*-value < 0.05) were selected as markedly differentially expressed circRNAs. The microarray data produced in this study have been uploaded to the NCBI/GEO repository (Accession Number: GSE145610).

### Construction of the ceRNA (circRNA-miRNA-mRNA) regulatory network

Several online bioinformatics platforms were used in conjunction to predict circRNA-miRNA-target gene associations. The targeted miRNAs and corresponding miRNA response elements of circRNAs were first obtained by utilizing a homemade miRNA target prediction tool derived from Arraystar. In addition, we extracted differentially expressed DE miRNAs from GSE35490, which included 16 TOF and 8 healthy control samples[18]. Target genes of integrated miRNAs were detected by targetScan (http://www.targetscan.org/vert_72/), miRTar-Base (http://mirtarbase.mbc.nctu. edu.tw/php/index.php) and miRDB (http://mirdb.org/) [20]. The circRNA-miRNA-mRNA regulatory network was constructed utilizing the integration of circRNA-miRNA pairs and miRNA-mRNA pairs. Finally, the regulatory network of TOF was visualized using the R package “ggalluvial” [21].

### Functional enrichment analyses

Gene Ontology (GO) and pathway enrichment analyses of genes involved in the ceRNA network were performed using the database for integrated discovery bioinformatics resources (Enrichr, http://amp.pharm.mssm.edu/Enrichr/enrich) [22]. Genes involved in the ceRNA network that were more relevant to CHD were identified. The comparative toxicogenomics database (http://ctdbase.org/) was used to integrate chemical-gene, chemical-disease, and gene-disease interactions to predict novel associations and generate expanded networks [23]. The relationships between gene products and congenital heart defects were analysed through these data. Here, relationships between genes extracted from the ceRNA network and diseases are shown in a radar chart.

### Statistical analysis

Data are presented as the mean ± standard deviation. All statistical data were analysed using the Statistical Program for Social Sciences (SPSS) 23.0 software (SPSS, Chicago, IL, USA). Comparisons between different groups were performed using independent Student’s t-test (two-tailed) or one-way ANOVA followed by Tukey’s post hoc test [24]. Pearson’s correlation analysis was used to identify the relationships between circRNAs and mRNAs [25]. In all cases, a P-value < 0.05 was considered to be a statistically significant difference.

## Results

### Screening of differentially expressed circRNAs

**Fig. 1:**
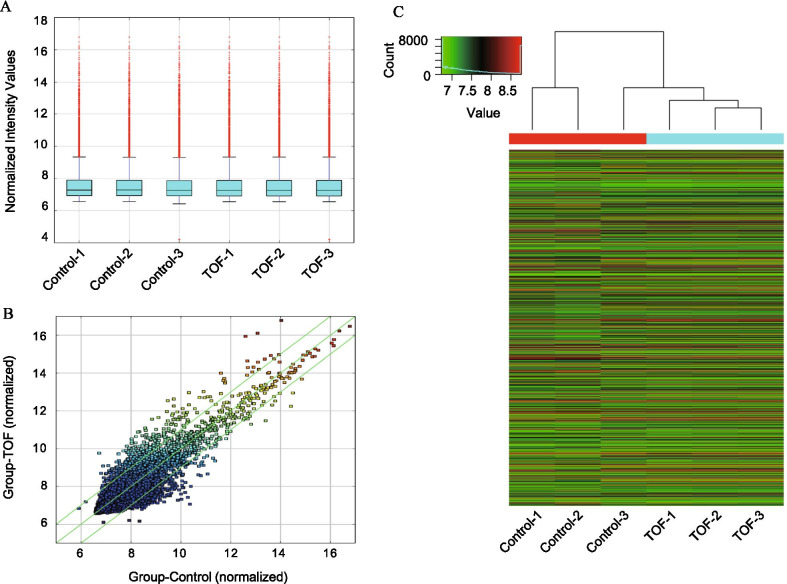
Overview of circRNA expression profiles. **a** A box plot was used to compare the distributions of circRNA expression values for TOF heart tissues and normal heart tissues (control) after normalization. **b** Scatter-plot representing variations in circRNA expression between the TOF group and control group (log2 scaled). **c** Unsupervised hierarchical clustering of dysregulated circRNA expression among samples. The colour scale shows the relative expression levels of circRNAs across different samples

We performed Arraystar Human circRNA Array analysis to identify TOF-related circRNAs. Box plots were constructed to show the distribution of circRNA expression profiles. After normalization, the distribution of log2 ratios across different samples is displayed in Fig. 1a. We then used a scatterplot to identify differentially expressed circRNAs between the TOF group and the control group (Fig. 1b). Unsupervised hierarchical clustering analysis revealed a deregulated circRNA expression pattern in clinical samples (Fig. 1c).

**Fig. 2:**
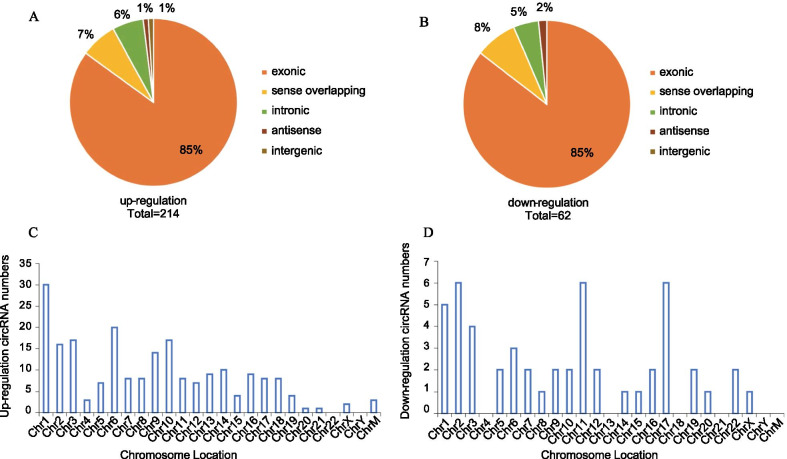
Annotation of differentially expressed circRNAs in TOF heart tissues. (**a,** **b**) Classification of dysregulated circRNAs. “Exonic” represents a circRNA arising from the exons of a linear transcript; “Intronic” represents a circRNA arising from an intron of a linear transcript; “antisense” represents a circRNA whose gene locus overlaps with the linear RNA but is transcribed from the opposite strand; “sense overlapping” represents circRNA originating from the same gene locus as the linear transcript. **c** The locations of upregulated circRNAs in human chromosomes. **d** The locations of downregulated circRNAs in human chromosomes

The general data, including the functional classifications and chromosome locations of these differentially expressed circRNAs, were summarized. Overall, 276 circRNAs were found to be significantly differentially expressed (|logFC|-value > 2.0, P-value < 0.05) (Additional file 1: Table 1). Compared to the control group, 214 circRNAs, including 183 exonic, 13 intronic, 16 sense overlapping, 1 antisense, and 1 intergenic circRNA, were upregulated, and 62 circRNAs, including 53 exonic, 3 intronic, 5 sense overlapping, and 1 antisense circRNA, were downregulated in the CHD group (Fig. 2a, b). As demonstrated in Fig. 2c and d, the up- and downregulated circRNAs were located on human chromosomes.

### The circRNA-miRNA-mRNA network in TOF

**Fig. 3:**
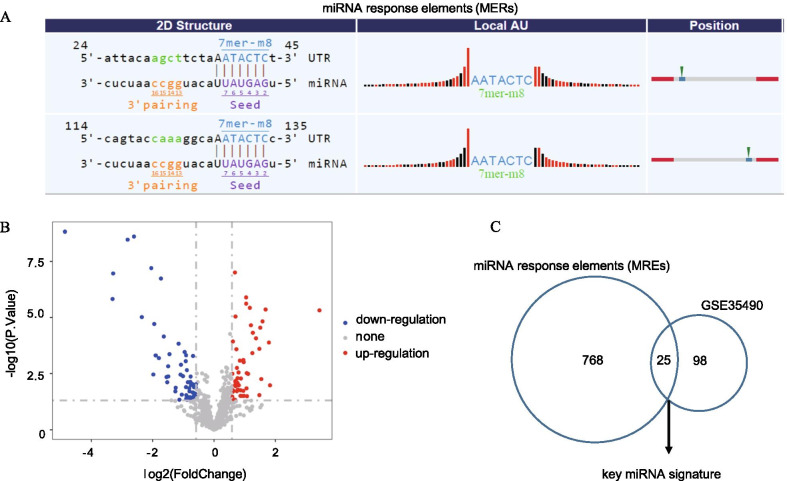
Identification of a 25 miRNA-based signature. **a** Results of target prediction and sequence analyses of microRNA response elements (MREs). The 2D structure shows the MRE sequence and the corresponding target miRNA seed type. **b** The volcano plot indicates the differentially expressed miRNAs obtained from GSE35490. The vertical lines correspond to 1.5-fold up and down, respectively, and the horizontal line across the screen represents *P* = 0.05. **c** The Venn diagram shows the numbers of key miRNA signatures

A total of 793 miRNA response elements (MREs) of the differentially expressed circRNAs were identified by utilizing the miRNA target prediction tool derived from Arraystar (Fig. 3a). Next, to consolidate the identified MREs that are associated with TOF and screen the more significant miRNAs, we used GSE35490 from the NCBI/GEO database to screen 123 differentially expressed miRNAs in the TOF group compared with the healthy groups (|logFC|-value > 1.5, *P *value < 0.05) (Fig. 3b). Using Venn diagram analysis, 25 shared miRNAs (key miRNA signature) were obtained and are presented in the Venn diagram (Fig. 3c).

**Fig. 4:**
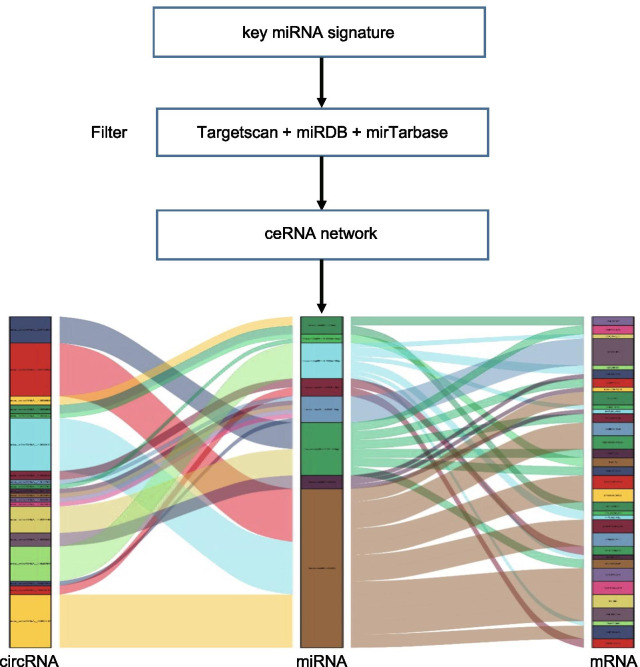
A Sankey diagram showing the ceRNA network in TOF. Each rectangle represents an element (circRNA, miRNA, mRNA), and the connection degree of each element is indicated based on the size of the rectangle

To predict the key target mRNAs, we further uploaded the abovementioned 25 key miRNAs to the miRDB, miRTarBase, and TargetScan databases for searching. Thirty-four key mRNAs that interacted with 8 of the 25 key miRNAs in all 3 datasets were selected. After removing the remaining 17 key miRNAs and the corresponding circRNAs, 19 circRNAs, 8 miRNAs, and 34 mRNAs were ultimately obtained to construct the ceRNA network related to TOF (Fig. 4; Additional file 2: Table 2).

### Functional GO terms and pathway enrichment analyses

**Fig. 5:**
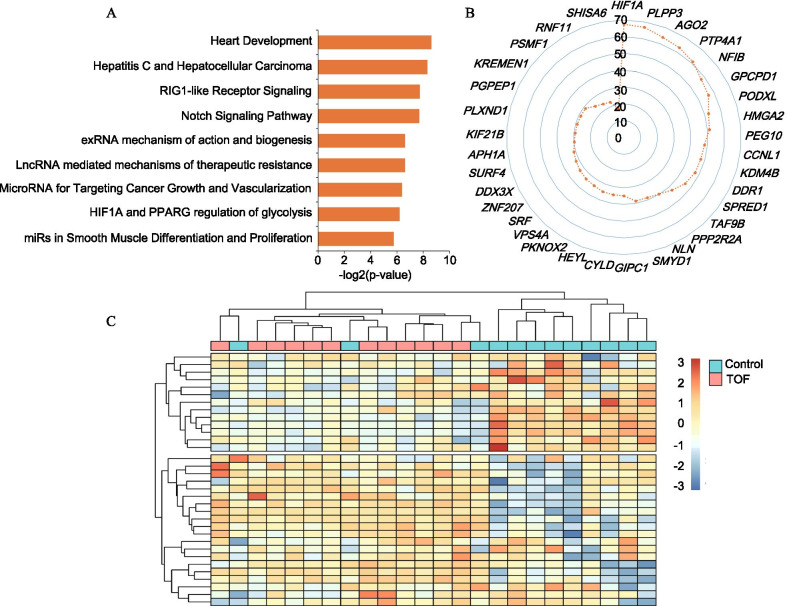
GO enrichment and pathway analyses of the key genes involved in the ceRNA network. **a** The top 9 significantly enriched functional GO terms retrieved using Enrichr. **b** Radar diagram depicting the relationships of congenital heart defects related to the key genes based on the CTD database. **c** Unsupervised clustering of samples based on the expression levels of the key genes

To characterize the functional consequences of our identified genes, we performed an enrichment analysis of the ChIP-X (ChEA) database with Enrichr for all 34 key genes involved in the ceRNA network. The results revealed that the GO term with the most statistical significance was heart development (P-value = 0.002538) (Fig. 5a).

In addition, we characterized the putative pathogenic genes of TOF based on the CTD database. A total of 34 genes had curated or inferred associations with congenital heart defects ranked by inference score. Among these genes, *HIF1A* was the most significant marker gene (Fig. 5b), as confirmed by three studies [11, 26, 27]. To make our results more reliable, we further assessed the association of 34 genes with TOF using the public database GSE35776 [28]. Unsupervised clustering resulted in two distinct subgroups according to the gene expression model (Fig. 5c).

### Upregulation of hsa_circ_0007798 expression in TOF heart tissues

**Fig. 6:**
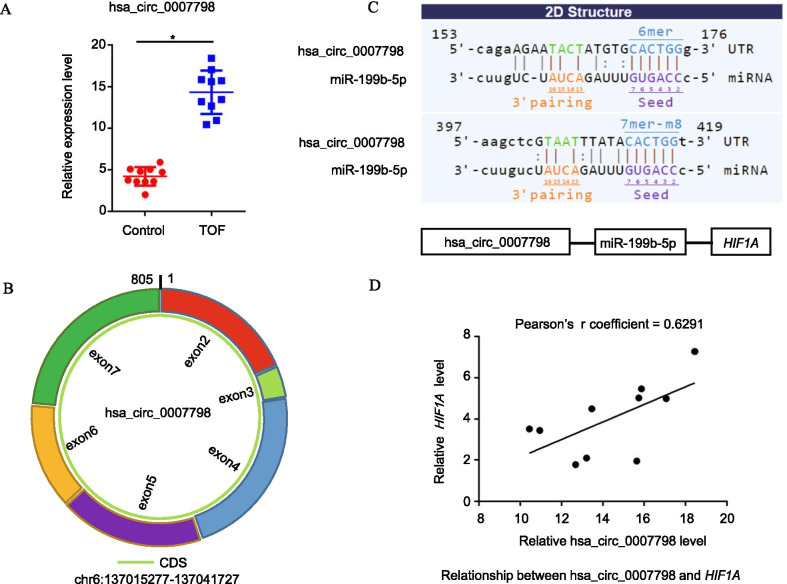
Functional hsa_circ_0007798/miR-199b-5p/*HIF1A* regulatory module. **a** Validation of the differential expression of hsa_circ_0007798. The expression level was analysed using qPCR (TOF: n = 10, Control: n = 10). **b**, **c** Predicted miRNAs targeting hsa_circ_0007798 based on a homemade tool derived from Arraystar’s software. **d** Validation of the relationship between hsa_circ_0007798 and *HIF1A*. The horizontal axis represents the expression of the mRNA, and the vertical axis indicates the expression of the circRNA

To validate the previous study results, we expanded the sample size. The expression levels of hsa_circ_0007798 in 10 TOF heart tissues and 10 healthy heart tissues were measured by qPCR, which revealed that hsa_circ_0007798 expression was significantly upregulated in the TOF group (P < 0.001, Fig. 6a). By inquiring from circBase (http://www.circbase.org), we knew that hsa_circ_0007798 is located on chromosome 6 and is composed of 6 exons containing the full 805-bp sequence (Fig. 6b). According to ceRNA theory, circRNAs mostly act by competitively sponging miRNAs to regulate the expression of their target genes. Therefore, based on our screening strategy, we first considered hsa_circ_0007798/miR-199b-5p/*HIF1A* (Fig. 6c). In addition, the results of the qPCR correlation analysis verified the positive linear relationship between hsa_circ_0007798 and *HIF1A* (Pearson r coefficient = 0.6291) (Fig. 6d). These results could make it possible for us to understand how predicted and screened key circRNA-miRNA-mRNA relationships are related to TOF progression.

## Discussion

It is well known that cardiac development is a dynamic and complex process. At the same time, cardiac development is finely regulated by the body itself, and minor developmental disorders (such as genetic or environmental factors) are sufficient to cause severe cardiac defects and subsequent embryonic or foetal death. TOF, as one of the most common cyanotic congenital heart diseases in the world, is estimated to account for 7%–10% of CHD cases [37] and is the result of the intersection of genetic, apparent, and environmental risk factors [27]. Studies have shown that rare extracardiac lesions in CNV and syndromic TOF may affect the reproductive suitability and propagation pattern of TOF [38], such as the 22q11.2 deletion and the associated 22q11.2 deletion syndrome [39]. Like other types of CHD, the exact cause of TOF is unclear. At present, with the help of new auxiliary treatment equipment, most children with TOF undergoing corrective surgical repair can survive to adulthood, but the long-term course of postoperative residual lesions in some children may still eventually lead to right ventricular dysfunction, ventricular arrhythmia and advanced sudden cardiac death [40, 41], which assumes a long-term economic burden on families and society. Studies have shown that TOF can occur in other noncardiac (syndromic) or isolated (nonsyndromic) environments. Among them, syndrome-type TOF accounts for approximately 20% of cases; it is mainly associated with 22q11.2 deletion syndrome (22q11del), and its clinical outcome has a worse prognosis [42, 43]. In a large study involving nine institutions, the most frequent harmful mutation in adults and infants with TOF was the *NOTCH1* mutation, which is estimated to account for 4.5% of nonsyndromic TOF cases. The next most common harmful mutation site was *FLT4*, which accounted for 2.4% of cases. In addition, previously involved heart transcription factors (such as *TBX1*, *NKX2.5*, *GATA4*, *HAND2*, and *GATA6*, etc.) leading to pathogenic mutations exist in only 1.2% of the population [44], and the lack of efficient biomarkers and the unclear mechanisms underlying TOF are still challenges in the field of cardiology [5, 6, 27].

Previous research has demonstrated that genetic and epigenetic mechanisms, including mutation, histone modification, DNA/RNA methylation, ncRNA modifications and others, play vital roles in cardiac development [8, 12, 18]. Among these major events, ncRNAs may be potential biomarkers for better prognosis and diagnosis. However, research into circRNAs is just beginning. Studies have found that circRNAs are produced by reverse splicing of the precursor mRNA (pre mRNA) of exons of thousands of genes in eukaryotes, in which the downstream 50 splice site (Ss) is linked to the upstream 30 Ss, and the resulting RNA loop is linked by a 30–50 phosphodiester bond at the linkage site[46, 47]. As early as 25 years ago, researchers found only a few circRNAs, which were generally considered aberrant splicing by-products with little functional potential[45, 49]. circRNAs are more stable than linear ribonucleic acids because circRNAs lack a free end, and their cyclic structures prevent degradation by nucleic acid exonuclease[48].

Rapid advances in biochemical methods and the use of high-throughput sequencing technology in recent years have served to isolate and identify a wider range of circRNAs [51]. Genome-wide analysis showed that most of the circRNAs were abundant and conserved across species, displaying cell type-, tissue-, and developmental stage-specific expression patterns in eukaryotic cells, indicating that circRNAs played a vital role in the regulation of transcriptomics and biological processes, including heart-related diseases [29–31].

Studies have shown that circRNAs are linked to the physiological and pathological development of many organisms, while some circRNAs are related to neuronal function, congenital immune response, cell proliferation, and pluripotency [53]. It has been found that circRNAs have various functions. At the molecular level, they participate in gene expression by isolating microRNA or protein, regulating RNA polymerase II (POL II) transcription, processing pre-interfering mRNA, and translating the resulting polypeptides [50]. Increasing evidence shows that circRNAs, as the binding sites for sponge RNAs and miRNAs competing for miRNAs, maintain RNA-binding protein and control the expression of alternative splicing and parental genes, indicating that cyclic RNA is becoming an important regulatory element at the transcriptional and posttranscriptional level [32, 52]. At present, competitive endogenous RNA (ceRNA) is a new field of RNA biology that implicates a large-scale regulatory network among multiple types of RNA (circRNAs, mRNAs, and miRNAs) at the transcription level [17]. A study has shown that circRNAs derived from *PWWP2A* can serve as endogenous miR-223 sponges to inhibit myocardial hypertrophy and heart failure [54]. The myocardial infarction-related circRNA (MICRA) level was found to be a robust predictor of LV dysfunction 3–4 months after myocardial infarction [55].

To date, this study is the first to provide systematic profiling of circRNA expression in TOF and to investigate further characterization of the role of the ceRNA network in the pathogenesis of TOF in the early life stage. Moreover, we identified and screened 214 significantly upregulated and 62 downregulated circRNAs through microarray analysis. Then, 19 circRNAs were chosen according to the established multistep screening strategy, and the circRNA-miRNA-mRNA regulatory module was constructed in an attempt to better explore TOF occurrence and development. We analysed the functions and pathways of key mRNAs participating in the ceRNA network using ChIP-X (ChEA) and the CTD database. These key mRNAs were mainly enriched in functions of “heart development” and “NOTCH signalling pathway”, which are closely associated with TOF [8]. In addition, we found that *HIF1A* is closely linked to human CHD, consistent with previous studies [33]. Studies have suggested that metabolic cardiac remodelling is associated with *HIF1A* deficiency and maternal diabetic exposure. The combination of diabetic pregnancy and *HIF1A* deficiency changes vascular homeostasis in the myocardium due to maternal diabetes and increases the risk of cardiovascular abnormalities in offspring [56]. In addition, reduced Nkx2-5 expression together with a sustained hypoxia-inducible factor 1α response led to embryo death [57].

This study has demonstrated that circRNAs can affect the occurrence and development of heart disease and cardiovascular disease, have potential as diagnostic or predictive biomarkers of disease, and offer new potential therapeutic targets. we rescreened the most promising ceRNA module by targeting HIF1A and performed correlation analysis to reveal that hsa_circ_0007798 has the positive correlation with HIF1A. Among the module, miR-199b-5p serves as a bridge between the circRNA and the mRNA. Coincidentally, miR-199b-5p was identified to participate in left ventricular remodeling, associated with pathologic cardiac hypertrophy [35-37]. These studies are in accordance with our finding showing hsa_circ_0007798/miR-199b-5p/HIF1A regulatory module is associated with the cardiac defects, including the TOF. However, compared with encoded RNAs and other noncoding RNAs (miRNAs and lncRNAs), our current understanding of circRNAs still has a significant gap, and the expression of circRNAs in human TOF myocardial tissue has not yet been reported. In this study, the Arraystar human circRNA chip was used to detect the expression profiles of circRNAs in TOF myocardial tissue and normal myocardial tissue, to analyse the differentially expressed circRNAs, to construct the ceRNA regulatory network of TOF using bioinformatics methods, to explore the possible pathogenesis of TOF without a genetic background, and to provide a new method for preventing the formation and development of TOF.

## Conclusions

In summary, we established a ceRNA network mediated by differentially expressed circRNAs in TOF based on the “ceRNA hypothesis”. This study provides new insight for mechanistic investigations and may offer candidate diagnostic biomarkers or potential therapeutic targets for TOF. In addition, we propose that the hsa_circ_0007798/miR-199b-5p/*HIF1A* signalling axis regulates TOF progression. Further studies are still required to validate the sponge effects of specific circRNAs, such as hsa_circ_0007798.

## Supplementary Information

**Additional file 1.Table 1** Differentially expressed circRNAs between the TOF group and the healthy control group.

**Additional file 2.Table 2** ceRNA regulatory modules.

## Data Availability

The microarray data produced in this study have been uploaded to the NCBI/GEO repository, and all data generated or analysed during this study are included in this published article.

## Abbreviations

CHD: Congenital heart disease
TOF: Tetralogy of Fallot;
circRNA: Circular RNA
VSD: Ventricular septal defect
ncRNAs: Noncoding RNAs
LncRNAs: Long ncRNAs
*HIF1A*: Hypoxia-inducible factor 1-alpha
ceRNAs: Competing endogenous RNAs
MREs: MiRNA response elements
qPCR: Quantitative PCR
GO: Gene Ontology
SPSS: Statistical Program for Social Sciences
pre mRNA: Precursor mRNA
Ss: Splice site
POL II: RNA polymerase II
RBP: RNA binding protein
MICRA: Myocardial infarction-related circRNA
